# Knowledge, attitude and adherence to COVID-19 prevention among community health workers in Nigeria

**DOI:** 10.11604/pamj.2022.42.307.30791

**Published:** 2022-08-24

**Authors:** Oluyemi Peter Atibioke, Fatimah Adepoju-Olajuwon, Oluwaseun Ayoola Ojomo, Adeyemi Olalekan Oladeji, Oluwakemi Bolatito Oripeloye, Kehinde Adesola Osinowo, Ademola Johnson Ajuwon, Oladapo Alabi Ladipo

**Affiliations:** 1 Association for Reproductive and Family Health (ARFH), Ibadan, Nigeria,; 2 Association for Reproductive and Family Health (ARFH), Abuja, Nigeria,; 3 Department of Health Promotion and Education, Faculty of Public Health, College of Medicine, University of Ibadan, Ibadan, Nigeria

**Keywords:** COVID-19, knowledge, attitude, adherence, anxiety, demographic variables

## Abstract

**Introduction:**

community health workers play important roles in curtailing the spread of COVID-19. This study therefore investigated the knowledge, attitude and adherence to practice of COVID-19 prevention-protocols among community health workers in selected States of Nigeria.

**Methods:**

purposive sampling method was adopted. A cohort of community health workers testing and enrolling human immunodeficiency virus (HIV) positive-clients into care were involved in the study. Questionnaire on Microsoft forms was completed by 366 participants. Data were analyzed using descriptive and inferential statistical methods.

**Results:**

key findings revealed that 87.80% have good knowledge of COVID-19; 96.10% positive attitude towards COVID-19 prevention-protocols and 97.20% adhere to the protocols. Demographics variables have significant positive effect on adherence to COVID-19 prevention-protocol among the respondents as follows: marital status (X^2^: 21.91; p: <0.05), gender (X^2^: 9.01; p: 0.003), ethnic group (X^2^: 17.45; p: <0.05), State of residence (X^2^: 32.51; p: <0.05), education status (X^2^: 18.44; p: 0.005). Findings revealed there is no significant relationship between knowledge of COVID-19 and the anxiety status of community health workers (p=0.90). There is positive relationship between knowledge of COVID-19 and attitude to guidelines and adherence to COVID-19 prevention-protocols. R=0.20 (<0.05) and 0.195 (<0.05) respectively.

**Conclusion:**

the high knowledge of COVID-19, positive attitude and adherence to the prevention-protocols among community health workers provides assurance of their ability to provide factual information to the community and their ability to promote good attitude and adherence to the prevention-protocols. Key sociodemographic variable like marital status, gender, ethnic groups, educational status and State of residents play significant roles in adherence to COVID-19 prevention-protocols.

## Introduction

The outbreak of severe acute respiratory syndrome coronavirus-2 (SARS CoV-2), the virus that causes COVID-19, was first reported by Chinese authorities in Wuhan, China at the end of December 2019 [1]. Since then, the virus has spread to virtually all countries of the world. In March 2020, the World Health Organization declared the COVID-19 a pandemic [2]. Nigeria reported her index case on 27^th^ February, 2020 and within three months, the virus has been reported in all the 36 States of the country including Abuja, the federal capital territory [3]. Health workers have important roles to play in controlling the spread of the virus including educating citizens on how to prevent the virus and care for patients. Health workers may be exposed to occupational hazards that put them at risk of disease, injury and even death in the context of the COVID-19 response. These occupational risks include: a) infections with COVID-19; b) skin disorders and heat stress from prolonged use of personal protective equipment (PPE); c) exposures to toxins because of increased use of disinfectants; d) psychological distress; e) chronic fatigue; and f) stigma, discrimination, physical and psychological violence, and harassment [4].

Health workers are expected to demonstrate adequate knowledge, be imbued with positive attitude, and adhere to the non-pharmaceutical interventions if they are to play the critical role of prevention and control of COVID-19. Previous studies have focused on online survey of facility-based health workers including doctors, nurses, and laboratory scientists in Nigeria. Few have focused on knowledge, attitude and practice of COVID-19 among community health workers (CHW) who, as frontline service providers, have the responsibility of entering hard-to-reach communities to provide healthcare services to community members which make them more vulnerable to contracting COVID-19. These frontline health workers provide essential services to community members especially COVID-19 prevention information, adherence to the use of PPE and referral for those who may have symptoms. They were also involved in HIV testing services, adherence counselling to patients on antiretroviral therapy (ART) and home-based ART deliveries for those on treatment who might have been denied access to ART refill due to COVID-19 lockdown. We report in this article results from a survey that assessed knowledge, attitude and adherence to COVID-19 prevention among community health workers in Nigeria. The study also assessed the COVID-19 related anxiety status in this population.

**Study objective:** to assess the knowledge, attitudes, and practices as well as anxiety status of community health workers towards COVID-19.

## Methods

**Study design:** study was a national cross sectional descriptive survey. Data were collected with the use of structured questionnaire using the Microsoft form containing two sections. The section A of the questionnaire elicited respondents´ demographic information, including their gender, age, marital status, ethnicity, educational qualification, religion, duration of practice as health worker and source of COVID-19 information while the part B assessed respondents´ COVID-19 knowledge, attitude and practice. The part B also contained assessment of anxiety level with 7 Items questionnaire adapted from Generalized Anxiety Disorder (GAD) scale [5]. The questionnaire was pilot tested among selected community health workers that have similar characteristics with population of interest and the validity of the questionnaire including the difficulty level were determined after the pilot test. Appropriate revisions were made on the questionnaire to ensure clarity and understanding of the question items.

**Study population:** the study comprised of CHW charged with the responsibility of entering hard-to-reach communities to provide healthcare services to community members using door-to-door approach. Key services delivered by this category of health workers within the community of coverage include: community mobilization, education and referral for uptake of important healthcare service; testing of vulnerable populations for HIV using sexual network testing and other targeted testing approaches; working with traditional birth attendants and faith based prayer homes to test pregnant women for HIV; assisted referrals to enroll HIV positive clients for ART uptakes. The outbreak of COVID-19 pandemic conferred additional responsibility on CHW including education on prevention of infection, testing, referral and use of personal protective equipment (PPE).

**Study setting:** the study covered 63 local government areas (LGAs) selected from 11 States in three regions of Nigeria namely Benue and Niger (North Central), Abia, Enugu, Imo (South East), Bayelsa, Delta, Edo, (South South) and Ogun, Osun and Oyo (South West). The States and LGAs were purposively selected based on their HIV burden and unmet needs defined as those who intend to know their HIV status but do not have access to HIV counselling and testing [6].

**Procedures for questionnaire administration:** the questionnaire was developed using Microsoft form and sent to all the 374 eligible CHW through their mobile phone using their WhatsApp number accessed from their database. Only 360 of them responded, yielding a response rate of 97%. The data were collected in April, 2020 during the pick of COVID-19 pandemic in Nigeria.

**Ethical considerations:** all ethical considerations in line with the principles laid down in the Declaration of Helsinki [7] were strictly followed. Prior to completing the questionnaire, the purpose and procedures for the study was explained to study participants. Information was also provided that participation in the study was voluntary, that data collected will be kept confidential and used for research purposes only. To this end, personal identifiers were not collected. Informed consent was assumed by participants who completed and sent back their questionnaire.

**Data management and analysis:** completed questionnaires were exported into excel spreadsheet and then subsequently exported into Statistical Package for the Social Sciences (SPSS) (version 20) for further cleaning and analysis. Participant´s demographic variables, COVID-19-related knowledge, attitude towards prevention information and actual practice of the prevention protocols, their anxiety status was analyzed using frequencies, percentages, means, and standard deviations. Correlation analyses was performed to understand the relationship between demographics, knowledge, attitude and practice of COVID-19 and their anxiety status. There were fifteen questions to assess the level of knowledge; ten to assess attitude and another fifteen to assess adherence to the practice of COVID-19 prevention protocol by the community health workers. Each question was scored as “1” for a correct response and “0” for an incorrect response. The maximum score obtainable by a respondent for knowledge and practice was 15 while the maximum score obtainable for attitude was 10. The average score for knowledge and practice was therefore set as “7.5” and 5 for attitude. Therefore, scores of 8 and above were classified as “good knowledge” and “good adherence” to COVID-19 prevention protocol while 6 and above was set as positive attitude towards the practice of the prevention protocol. However, score less than 8 was classified as “poor knowledge”, “negative attitude” and “poor adherence”.

The categorization of the 7-item generalized anxiety disorder scale was done as follows: “0”= not at all; “1”= several days; “2”= more than half the days; “3”= nearly every day. The maximum obtainable score for each respondent was “21” and the higher the score, the higher the level of COVID-19 induced anxiety. Levels of anxiety of “0-4” was classified as “minimal anxiety”, “5-9” as “mild anxiety”, “10-14” as “moderate anxiety” and any score from “15-21” was classified as “severe anxiety”.

## Results

Table 1 shows the demographic characteristics of respondents. Majority of participants 29.4 and 31.1 were age 26-30 and 31 to 35 respectively. Less than half (49%) of the participants were never married. Also, 64.7% of them were female with more than half being from Igbo ethnicity. More of the CHWs were resident in Ogun State (14.2%); 12.2% were from Abia State; 11.4% from Oyo State; 11.2% from Delta State and only about 4% were resident in Niger State. Other demographic characteristics are presented in Table 1.

**Table 1:** demographic characteristics of ARFH counselor testers

Variables	Frequency	(%)	Variables	Frequency	(%)
**Age in years**			**Marital status**		
<20 years	2	0.6	Single	176	48.9
21 - 25 years	106	15.0	Married	169	46.9
26 - 30 years	112	29.4	Separated	2	0.6
31 - 35 years	56	31.1	Widowed	13	3.6
36 - 40 years	30	15.6			
>40 years		8.3			
**Gender**			**Ethnicity**		
Female	233	64.7	Hausa	52	14.4
Male	127	35.3	Igbo	185	51.4
			Yoruba	123	34.2
**State**			**Highest education level**		
Abia	44	12.2	Primary school - completed	2	0.6
Bayelsa	24	6.7	Secondary school completed	22	6.1
Benue	35	9.7	Certificate/diploma in clinical/health/pharmacy	20	5.6
Delta	40	11.1	NCE/OND	119	33.1
Edo	24	6.7	Registered nurse/midwife	3	0.8
Enugu	26	7.2	HND/BEd/BSc	177	49.2
Imo	35	9.7	Postgraduate education	17	4.7
Niger	14	3.9			
Ogun	51	14.2			
Osun	26	7.2			
Oyo	41	11.4			
**Religion**			**Duration of practice as community health worker**		
Christianity	313	86.9	Entry level	2	0.6
Islam	47	13.1	1-5 years	208	57.8
			6-10 years	65	18.1
			More than 10 years	23	6.4

NCE: Nigeria certificate in education; OND: ordinary national diploma; HND: higher national diploma; BEd: bachelor of education; BSc: bachelor of science

Table 2 presents information on knowledge of community health workers on COVID-19. A good number (97.2%) understood that coronavirus is a severe acute respiratory syndrome; 96.7% knew that COVID-19 virus could spread through contact with respiratory droplets of infected individuals; 98.3% avoiding crowded places such as train stations, social and religious gathering and avoid taking public transportation as a veritable way of avoiding COVID-19 infection. Also, 98.6% knew that isolation and treatment of people who are infected with the COVID-19 virus were effective ways to reduce the spread of the virus while 98.6% understood that timely diagnosis and adequate medical intervention for infected persons could facilitate survival of COVID-19. Unfortunately, 80.6% also believed that persons with COVID-19 could not infect others with the virus when a fever is not present. The participants also scored below 70% in all other knowledge parameters.

**Table 2:** level of knowledge of community health workers on COVID-19

Variables	Correct knowledge	Incorrect knowledge
Corona virus (COVID-19) is a severe acute respiratory syndrome	350 (97.2)	10 (2.8)
Is there currently an effective cure for COVID-19?	247 (68.6)	113 (31.4)
Will all persons with COVID-19 infection result in severe cases	204 (56.7)	156 (43.3)
Will eating or contact with wild animals result in the infection by the COVID-19 virus	194 (53.9)	166 (46.1)
Persons with COVID-19 cannot infect others with the virus when a fever is not present	290 (80.6)	70 (19.4)
The COVID-19 virus can spread through contact with respiratory droplets of infected individuals	348 (96.7)	12 (3.3)
To prevent the infection by COVID-19, individuals should avoid going to crowded places such as train stations, social and religious gathering and avoid taking public transportation	354 (98.3)	6 (1.7)
Isolation and treatment of people who are infected with the COVID-19 virus are effective ways to reduce the spread of the virus	355 (98.6)	5 (1.4)
People from Africa with hot weather are immune from contracting COVID-19	218 (60.6)	142 (29.4)
With timely diagnosis and adequate medical intervention, infected persons can survive COVID-19	355 (98.6)	5 (1.4)
Antibiotics kill COVID-19	215 (59.7)	145 (40.3)
Thermal scanners can diagnose COVID-19	96 (26.7)	264 (73.3)
Garlic, ginger and black fruits oil can cure COVID-19	179 (49.7)	181 (50.3)
COVID-19 is a virus from 5-G network	216 (60.0)	144 (40.0)
COVID-19 can be transmitted between animals and people	118 (32.8)	242 (67.2)

Table 3 shows the attitude of CHW towards COVID-19 guidelines. It can be seen that many of the CHW had positive attitude towards the laid down guidelines and principles. Findings revealed 94% restricted their movement as appropriate and practice social distancing, 81.7% believed COVID-19 is a death sentence, and 97.2% agreed to isolate themselves the moment they feel any COVID-19 symptoms. Generally, 96.1% of the CHWs had positive attitude and only about 3.9% had negative attitude to COVID-19 laid down guidelines and principles.

**Table 3:** attitude of community health workers towards COVID-19 laid down guidelines and principles

Variables	Positive attitude (%)	Negative attitude (%)
The restriction of movement, social distancing and complete lockdown by some States are necessary to stop the spread of COVID-19	338 (93.9)	22 (6.1)
COVID-19 is a death sentence?	294 (81.7)	66 (18.3)
I will isolate myself the moment I begin to feel any of the symptoms of COVID-19	350 (97.2)	10 (2.8)
It is better to pray against COVID-19 infection than observing some of the WHO recommended prevention which may not be too effective	284 (78.9)	76 (21.1)
When there is no visible dirt, there is no need to wash hands repeatedly	337 (93.6)	23 (6.4)
Avoiding touching my nose, mouth or eyes before washing my hands with soap after contact is a way of preventing the spread of COVID-19	340 (94.4)	20 (5.6)
Distancing myself from person infected with COVID-19 is not ideal to prevent stigma and discrimination?	125 (34.7)	235 (65.3)
Hugging a loved one with the suspected case of COVID-19 is a way of showing support to such individuals	334 (92.8)	26 (7.2)
A home visit should be provided to those infected with COVID-19	273 (75.8)	87 (24.1)
It is important to report any suspected case of COVID-19 to ministry of health official or NCDC	358 (99.4)	2 (0.6)

Level of adherence to practice of basic COVID-19 guidelines by CHW can be seen in Table 4. About 39% of CHW went to crowded places during the last one month, 21.1% did not wear a mask while leaving home for community activities, 39.4% did not sneeze or cough into their elbow rather they did into their hands, 1.7% did not wear gloves when testing for HIV, 12.2% had close contact with sick people and 1.9% touched their eyes, nose or mouth with unwashed hand. However, the overall level of adherence to practice of basic guidelines by CHWs is 97.2%.

**Table 4:** level of adherence to practice of basic guidelines on COVID-19 among community health workers of ARFH

Variables	Good adherence (%)	Poor adherence (%)
In the last one month, have you gone to any crowded place	221 (61.4)	139 (38.6)
In recent days, have you worn a mask when leaving home for community activities?	284 (78.9)	76 (21.1)
If I have the need to sneeze or cough, I do that by covering my mouths with my hands	142 (39.4)	218 (60.6)
I clean all surfaces before contact to prevent contact of COVID-19 from droplet on surfaces	335 (93.1)	25 (6.9)
I wear my gloves anytime I want to touch anyone for Testing	354 (98.3)	6 (1.7)
I avoid close contact with people who are sick	316 (87.8)	44 (12.2)
I avoid touching my eyes, nose, or mouth with unwashed hand	353 (98.1)	7 (1.9)
I frequently rub alcohol-based hand sanitizer with at least 60% alcohol	334 (92.8)	26 (7.2)
I always wash my hands anytime I have contact with surface or person	347 (96.4)	13 (3.7)
I avoid eating and drinking in the course of community work	321 (89.2)	39 (10.8)
I have put on hold all my social gathering activities to prevent the spread of COVID-19	341 (94.7)	19 (5.3)
I physically participated in church activities with more than 50 people in attendance in the last one week	315 (87.5)	45 (12.5)
I listen to daily update on COVID-19 and adhere to all the prevention information stipulated by WHO and NCDC	352 (97.8)	8 (2.2)
I avoid conducting an HIV test for all individuals that are very sick with symptoms of COVID-19	230 (63.9)	130 (36.1)
I have the telephone contact of my supervisor to contact in case of any identification of COVID-19 suspect	342 (95.0)	18 (5.0)

**Figure 1:**
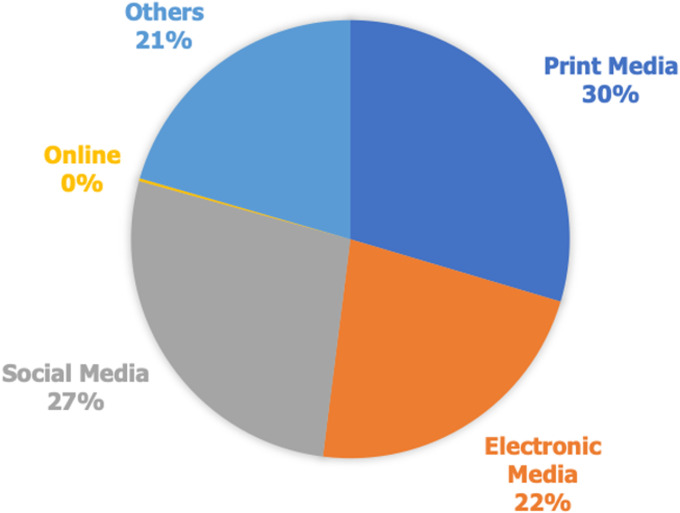
major source of COVID-19 information for community health workers

Also, Table 5 considered the relationship between socio-demographic variables and adherence to COVID-19 prevention protocols. Finding revealed some of the demographics (marital status, gender, ethnic group, State of residence, highest education attained) have significant positive effect on the adherence level of the community health workers as follows; marital status (X^2^: 21.91; p: <0.05), gender (X^2^: 9.01; p: 0.003), ethnic group (X^2^: 17.45; p: <0.05), State of residence (X^2^: 32.51; p: <0.05), education status (X^2^: 18.44; p: 0.005) indicated positive effect on the adherence level of the community health workers are significant factor that showed that more attentions are needed in enlightening the less educated people. However, age group, duration of practice as CHW and religious belief did not show any effect on the adherence to practice of COVID-19 prevention protocols. The major source of COVID-19 information for community health workers is indicated in Figure 1. It can be found that the sources of information were not limited to one but a combination of sources which are mostly through print media (newspaper, research articles, magazine etc.); electronic media (television and radio) and social media (WhatsApp, Facebook, Instagram, Twitter etc.) and others (families, friends, colleagues etc.). The percentage of use of each of the sources of information is presented in Figure 1.

**Table 5:** effect of demographics on adherence to practice of by community health workers to NCDC guidelines

Variables	Poor adherence (%)	Good adherence (%)	X^2^ (p-value)
**Age in years**			
Below 20 years	0 (0.0)	2 (0.6)	2.15 (0.83)
21 - 25 years	3 (30.0)	51 (14.6)	
26 - 30 years	2 (20.0)	104 (29.7)	
31 - 35 years	3 (30.0)	109 (31.1)	
36 - 40 years	1 (10.0)	55 (15.7)	
Above 40 years	1 (10.0)	29 (8.3)	
**Marital status**			
Single	8 (80.0)	168 (48.0)	*21.91 (<0.05)
Married	1 (10.0)	168 (48.0)	
Separated	1 (10.0)	1 (0.3)	
Widowed	0 (0.0)	13 (3.7)	
**Gender**			
Female	2 (20.0)	231 (66.0)	*9.01
Male	8 (80.0)	119 (34.0)	(0.003)
**Ethnicity**			
Hausa	6 (60.0)	46 (13.1)	*17.45 (<0.05)
Igbo	3 (30.0)	182 (52.0)	
Yoruba	1 (10.0)	122 (34.9)	
**State of residence**			
Abia	1 (10.0)	43 (12.3)	*32.51 (<0.05)
Bayelsa	1 (10.0)	23 (6.6)	
Benue	6 (60.0)	29 (8.3)	
Delta	0 (0.0)	40 (11.4)	
Edo	0 (0.0)	24 (6.9)	
Enugu	1 (10.0)	25 (7.1)	
Imo	0 (0.0)	35 (10.0)	
Niger	0 (0.0)	14 (4.0)	
Ogun	0 (0.0)	51 (14.6)	
Osun	0 (0.0)	26 (7.4)	
Oyo	1 (10.0)	40 (11.4)	
**Education level**			
Completed primary school	1 (10.0)	1 (0.3)	*18.44 (0.05)
Completed secondary school	0 (0.0)	22 (6.3)	
Certificate/diploma in clinical/health/pharmacy/laboratory/management	0 (0.0)	20 (5.7)	
Registered nurse/midwife	0 (0.0)	3 (0.9)	
NCE/OND	4 (40.0)	115 (32.9)	
BEd/BSc/HND	5 (50.0)	172 (49.1)	
Postgraduate education	0 (0.0)	17 (4.9)	
**Religion**			
Christianity	10 (100.0)	303 (86.6)	1.55 (0.21)
Islam	0 (0.0)	47 (13.4)	
**Duration of practice as community health worker**			
Entry level	0 (0.0)	2 (0.6)	8.43 (0.08)
1-5 years	3 (30.0)	205 (58.6)	
6-10 years	1 (10.0)	64 (18.3)	
More than 10 years	1 (10.0)	22 (6.3)	

NCE: Nigeria certificate in education; OND: ordinary national diploma; HND: higher national diploma; BEd: bachelor of education; BSc: bachelor of science

The relationship between knowledge, attitude and practice on COVID-19 is shown in Table 6. There is positive relationship between knowledge of COVID-19 and attitude to guidelines as well as adherence to practice of COVID-19 prevention protocols. However, negative relationship exists between attitude to guideline and adherence to practice of guidelines.

**Table 6:** relationship between knowledge, attitude and practice on COVID-19

Variables	Correlation to Knowledge of COVID-19 (P-value)	Correlation to adherence to practice; (P-value)
Attitude to NCDC guideline correlation (P-value)	*0.20 (<0.05)	-0.18 (0.74)
Adherence to practice; correlation (P-value)	*0.195 (<0.05)	-

Majority of the participants (68.3%) had minimal anxiety, 19.4% had mild anxiety; 8.6% had moderate anxiety while only 3.6% had severe anxiety. Further analysis also revealed there is no significant relationship between knowledge of COVID-19 and the anxiety status of the community health workers (p=0.90) as shown in Table 7. The breakdown shows that out of a total of 246 CHWs with minimal anxiety, 88.6% had good knowledge of COVID-19; out of 70 CHWs with mild anxiety, 85.7% also had good knowledge. Furthermore, 87.1% of 31 with moderate anxiety and 84.6% out of 13 with severe anxiety had good knowledge of COVID-19 respectively.

**Table 7:** relationship between knowledge of COVID-19 and anxiety status of the community health workers

		Minimal anxiety (%)	Mild anxiety	Moderate anxiety	Severe anxiety	Total (%)	X^2^ (P-value)
Knowledge of COVID-19	Poor knowledge	28 (11.4)	10 (14.3)	4 (12.9)	2 (15.4)	44 (12.2)	0.574 (0.90)
	Good knowledge	218 (88.6)	60 (85.7)	27 (87.1)	11 (84.6)	316 (87.8)	
	Total	246 (68.3)	70 (19.4)	31 (8.6)	13 (3.6)	360	

## Discussion

This study assessed the level of knowledge, attitudes, and practices as well as anxiety status of community health workers towards COVID-19 in selected States of Nigeria. On the aggregate, there is 87.80% knowledge of COVID-19 among the community health workers. Many of the CHWs had good knowledge of the COVID-19 prevention and transmission in particular. Our finding is similar to some other findings in the previous studies on COVID 19. Puspitasari *et al*. 2020 in a meta-analysis of 7 knowledge-based studies among health workers across nations reported that 6 out of the 7 studies indicated good knowledge of COVID-19 [8].

Also, the attitudes and good practice of COVID-19 prevention guidelines was found encouraging amongst the community health workers. These findings were similar to those reported in a meta-analysis on knowledge, attitude and practice of COVID-19 that 5 out of the 7 studies reviewed showed positive attitude however, only 3 of the studies indicated good practices amongst its study population [8]. This exemplifies that more work is needed to be done to ensure maximum adherence to guidelines. Unfortunately, our findings are not in tandem with a study conducted in Bangladesh among adult which indicated a higher perception but low knowledge about contracting COVID-19 [9]. Adherence practice is a major aspect that require urgent intervention in any country amongst the citizens, this will help in mitigating the spread of the virus and any other diseases. The study also revealed that most of the information about COVID-19 by the participants were gotten from the combination of print media (newspaper, research articles, magazine etc.); electronic media (television and radio) as well as social media (WhatsApp, Facebook, Instagram, Twitter etc.) platforms. However, there is concern that only a few got information from training organized by fellow health workers. This is in agreement with a study in Yemen conducted amongst healthcare workers who cited that their sources of knowledge about COVID-19 are mostly from television and radio, followed by social media, and only few were peer-reviewed scientific articles and friends [10]. The need for health regulation bodies like WHO, Nigeria Centre for Disease Control (NCDC), ministry of health to step up their games and adopt the modern day communication especially social media in the sensitization of people on health issues has become more urgent than ever. The Ministry of Health at all levels in Nigeria in particular need to scale up the utilization of the social media especially in local languages of the people to increase knowledge of health education especially by non-English speakers. A study conducted in Ho Chi Minh City of China amongst health care workers (HCW) illustrated that HCWs are more interested in social media to gather knowledge on an emerging infectious disease like COVID-19 than the official website of the ministry of health at the present time (11). This is a significant concern for the government because it is essential to think about a diversity of channels to keep frontline health workers at all levels informed about knowledge and learning materials on this epidemic and, particularly, to communicate information to the minority of HCWs who do not have adequate knowledge or are not currently aware of any issue relating to COVID-19 [11].

The negative relationship that existed between the socio-demographics and knowledge of COVID-19, as well as the attitude of some selected CHWs to NCDC guidelines can be related to the fact that the CHWs are generally informed at same level no matter where they reside and operates from. However, some of the demographics (marital status, gender, ethnic group, state of residence, highest education attained) indicated positive effect on the adherence level of the community health workers are significant factor that showed that more attentions are needed in enlightening the less educated people, some vulnerable sets like the separated and the widowed as well as some ethnic groups amongst these second line health workers in order to avail them of any consequences of their ignorance. Proper education of the CHWs will have a wide effect on the community they serve on a continuous basis. The significant results observed across States could also be linked to the variations in the control measures put in place to curtail the spread of COVID-19.

Furthermore, the negative relationship between anxiety level of CHWs and knowledge of COVID-19 could be related to the confidence of many Nigerian that the COVID-19 cases is only widespread amongst the high and mighty, myths on weather condition, as well as trust in the cure with herbs which then affected their adherence to NCDC guidelines. According to a statement by Olapegba *et al*. from their preliminary assessment of COVID-19 knowledge and perception in Nigeria, Nigerians had reasonably good knowledge of the COVID-19 but are weighed down with numerous fallacies, some are relying on the theory of hot weather in Africa, consumption of gins, herbs and African foods as well as chloroquine and antibiotics as precautions to the spread of the pandemic [12].

**Limitations:** there are several limitations inherent in this study. One limitation is that the study was only conducted in selected States in Nigeria, and not all community health workers in the selected States participated in the study, which limits the generalizability of the findings. Also, limitation is related to the questionnaire; due to time factor and the urgency in sharing the questionnaires with the participants, it was only validated by few public health professionals. Thus, a more in-depth evaluation of the validity and reliability of the questionnaires may have produced a more robust tool.

## Conclusion

The finding of this study revealed there exist high knowledge of COVID-19 among community health workers which is very important in the control of spread of COVID-19 especially at community level. The community health workers are key human resource for health that are expected to provide basic information to community members on COVID-19 and how to curtail the spread. Continuous strengthening of public health knowledge for health care workers including community health workers at the grassroots on COVID-19 should therefore be sustained. This in effect, would change behaviors and activities towards COVID-19 at the community levels especially when the community members have access to factual information from the community health workers. Attitude and practice of COVID-19 prevention protocol was also found to be high. This implies that the CHW will be able to lead the community by example of positive attitude and good practice of COVID-19 prevention protocols. This is in line with the social learning theory which suggests that social behavior is learned by observing and imitating the behavior of others. The CHW are seen in the community as significant person in health decision making and their attitude and practice have potentials to influence the decision of community members on health. The role of demographic variables especially sex was also revealed in the study. Women have better knowledge that men which is a call for action that more attention should be focused on men the management of COVID-19. They women seem to have taken strategic position in public health practice while men are gradually left behind which could be counterproductive in future since the men still wield a lot of power in decision making. With adequate intervention and follow-up also, health care workers will adhere better to guidelines and take safety precautions more seriously so as to keep safe in the face of infectious diseases. The need to also provide safety materials was also brought to the fore in this study. Without adequate provision of personal protective equipment to front line health workers, they may not be motivated to take appropriate behaviour especially behaviour that will motivate them to provide adequate services to community members in pandemic situation. Provision of PPE will undoubtedly advance the rate of positive attitudes of front line health workers. In addition, it is essential that hospitals' website as well as the ministry of health´s be updated regularly and also encourage CHWs to access this channel for all health-related issues and information especially the social media.

### What is known about this topic

- Community health workers (CHWs) had good knowledge of the COVID-19 symptoms and transmission routes;
- Community health workers (CHWs) lacked knowledge regarding the infectiousness of asymptomatic patients.

### What this study adds

- Evidence that CHWs are key human resource for health that are expected to provide basic information to community members on COVID-19 and how to curtail the spread;
- Evidence that CHWs will be able to lead the community by example of positive attitude and good practice of COVID-19 prevention protocols.
